# Stewarding beyond the 9–5: Implementation of overnight review of rapid blood culture identification panel results by intensive care unit pharmacists

**DOI:** 10.1017/ash.2025.28

**Published:** 2025-02-17

**Authors:** Sarah M. Arduser Sindelar, Jonathan H. Ryder, Jeremy Tigh, Theodore Blum, Jessica Prucha, Paul Fey, Scott J. Bergman, Trevor C. Van Schooneveld, Shawnalyn W. Sunagawa

**Affiliations:** 1 Department of Pharmaceutical and Nutrition Care, Nebraska Medicine, Omaha, NE, USA; 2 Department of Internal Medicine, Division of Infectious Diseases, University of Nebraska Medical Center, Omaha, NE, USA; 3 Department of Pharmacy, Good Samaritan Regional Medical Center, Corvallis, OR, USA; 4 Department of Pathology, Microbiology, and Immunology, University of Nebraska Medical Center, Omaha, NE, USA; 5 Department of Pharmacy Practice and Science, College of Pharmacy, University of Nebraska Medical Center, Omaha, NE, USA

## Abstract

Rapid blood culture identification is most effective with antimicrobial stewardship feedback, which is limited during non-business hours. We implemented overnight review of Blood Culture Identification 2 panel results by intensive care unit pharmacists and demonstrated reduced time to evaluation (3.6 vs 9.3 hours, *P* < .01).

## Introduction

The BioFire® Blood Culture Identification 2 (BCID2) panel (BioMérieux) is a rapid diagnostic tool that detects bacterial and yeast pathogen genetic targets and antimicrobial resistance markers in positive blood cultures, providing organism identification within 1–2 hours.^1^ Rapid blood culture diagnostics in combination with antimicrobial stewardship program (ASP) interventions decrease time to optimization of antimicrobials, resulting in decreased mortality, shorter hospital lengths of stay, and lower costs.^2–6^ However, traditional ASP review typically occurs only on weekdays during business hours, potentially limiting these interventions.

In August 2021, Nebraska Medicine upgraded from the original BCID to the BCID2 panel and updated guidance for clinicians. ASP performs review of all blood culture results during business hours on weekdays. To optimize care for the most critically ill patients with bloodstream infections, we implemented a workflow in September 2022 for the overnight intensive care unit (ICU) clinical pharmacist to review BCID2 results in ICUs reported after ASP hours. The ICU pharmacists were selected since they provide 24-hour, 7-day-a-week on-site care to patients most likely to benefit from early appropriate antibiotic therapy. Thus, the objective of this study was to assess the impact of overnight ICU pharmacist review on time to evaluation of antimicrobials based on BCID2 results and accepted antimicrobial order recommendations based on our institutional ASP guidance.

## Methods

We conducted a single-center, quasi-experimental quality improvement study at an academic medical center licensed for 718 beds, including 96 ICU beds. Patients were included if they had a positive blood culture with a BCID2 result while in an ICU during overnight clinical pharmacist coverage (21:00–07:00) Monday–Friday (Supplemental Figure 1). We compared ASP review of BCID2 results from overnight August 1, 2021 to August 31, 2022 (pre-cohort) to overnight ICU pharmacist review from September 1, 2022 to September 30, 2023 (post-cohort). Patients were excluded if they transferred from outside hospitals with bloodstream infections, had recurrent bloodstream infections with the same organism, and patients discharged or deceased within 24 hours of BCID2 results. We chose to exclude BCID2 results between Friday after 17:00 and Monday before 7:00 for intervention comparison between our cohorts because these results would not always be reviewed routinely by weekday ASP.

The primary outcome was time to evaluation of therapy based on BCID2 result, defined as hours from BCID2 result to time of review documentation by ASP or overnight ICU pharmacists. The overnight ICU pharmacist reviewed BCID2 results per institutional workflow (Supplemental Figure 2) and provided recommendations in concordance with the ASP guidance for BCID2 results (Supplemental Material). All BCID2 results were reviewed; however, intervention occurred only when a change in therapy was recommended (Supplemental Figure 3). Secondary outcomes included number and types of documented interventions, accepted antimicrobial interventions, time to accepted antimicrobial order changes, types of accepted antimicrobial order changes, empiric antimicrobial concordance with final pathogen susceptibilities, 30-day all-cause mortality, and 30-day all-cause readmission. Time to accepted antimicrobial order changes were defined as time of ASP or overnight ICU pharmacist BCID2 review to time of antibiotic order placement.

Interventions were categorized as antimicrobial order changes, initiation of infectious diseases (ID) consults, or *Staphylococcus aureus* bacteremia consults. Institutional policy requires an ID consult for all patients with *Staphylococcus aureus* bacteremia. If not already consulted, this process is initiated by ASP or recommended by overnight ICU pharmacists. For antimicrobial order changes, effective empiric therapy was defined as administration of an antimicrobial to which the organism demonstrated *in vitro* susceptibility (ie, reported as susceptible per Clinical and Laboratory Standards Institute). Each study outcome was independently adjudicated by three investigators (S.W.S., J.H.R., J.T.), and our institutional review board deemed this project as quality improvement exempt from review. Descriptive statistics were performed for cohort characteristics. Primary and secondary outcomes were compared using independent sample *t*-tests, χ^2^, Fisher’s exact, and Mann-Whitney *U* tests.

## Results

There were 422 overnight BCID2 results from 395 patients included, 192 in the pre-cohort and 230 in the post-cohort. Table 1 summarizes cohort characteristics, including BCID2 organisms. There were no significant differences between the two groups. Infections were more frequently classified as community-onset (58.5%), defined as positive blood cultures drawn ≤ 48 hours of hospital admission, with *Staphylococcus epidermidis* (21.3%) and *Escherichia coli* (12.1%) detected most often. There were 41 (9.7%) polymicrobial BCID2 results and 32 (7.6%) had no targets detected on BCID2.

**Table 1.** Cohort characteristics

Characteristic	Overall (N = 422)	Pre-cohort (n = 192)	Post-cohort (n = 230)	*P*-Value
Age (years), mean ± SD	57.9 ± 18.1	56.9 ± 18.5	58.9 ± 17.8	.256
Gender, male, n (%)	243 (57.6)	112 (58.3)	131 (57.0)	.776
Hospital length of stay (days), median (IQR)	13.9 (7.1, 32.8)	13.7 (7.4, 33.6)	14.4 (7.4, 33.4)	.558
Immunocompromised^a^, n (%)	149 (35.3)	66 (34.4)	83 (36.1)	.714
Infection type^b^, n (%)				.472
Community-onset	247 (58.5)	116 (60.4)	131 (56.9)	
Hospital-acquired	175 (41.5)	76 (39.6)	99 (43.1)	
Organisms Detected on BCID2, n (%)				.497
* Staphylococcus epidermidis*	90 (21.3)	43 (22.4)	47 (20.4)	
* Escherichia coli*	51 (12.1)	23 (12)	28 (12.2)	
* Staphylococcus aureus*	50 (11.8)	24 (12.5)	26 (11.3)	
Polymicrobial	41 (9.7)	21 (10.9)	20 (8.7)	
None detected	32 (7.6)	11 (5.7)	21 (9.1)	
* Staphylococcus* spp. only	28 (6.6)	17 (8.9)	11 (4.8)	
* Streptococcus* spp. only	25 (5.9)	10 (5.2)	15 (6.5)	
* Klebsiella pneumoniae* group	17 (4.0)	9 (4.7)	8 (3.5)	
* Enterococcus faecium*	15 (3.6)	7 (3.7)	8 (3.5)	
* Pseudomonas aeruginosa*	12 (2.8)	6 (3.1)	6 (2.6)	
Other^c^	61 (14.6)	21 (10.9)	40 (17.4)	
Contaminants^d^, n (%)	127 (30.1)	65 (33.9)	62 (27.0)	.124

Abbreviations: BCID2, Blood Culture Identification 2; SD, standard deviation; IQR, interquartile range.

a Solid organ transplant, bone marrow transplant, hematology/oncology, human immunodeficiency virus (HIV)/acquired immunodeficiency syndrome (AIDS).

b Community-onset = positive blood cultures drawn ≤ 48 hours of hospital admission; Hospital-acquired = positive blood cultures drawn > 48 hours of hospital admission.

c *Enterococcus faecalis* (n = 8), *Streptococcus pneumoniae* (n = 6), Enterobacterales order only (n = 6), *Klebsiella aerogenes* (n = 6), *Serratia marcescens* (n = 5), *Candida albicans* (n = 5), *Candida glabrata* (n = 5), *Streptococcus pyogenes* (n = 4), *Klebsiella oxytoca* (n = 4), *Enterobacter cloacae* complex (n = 3), *Staphylococcus lugdunensis* (n = 2), *Streptococcus agalactiae* (n = 2), *Acinetobacter baumannii complex* (n = 1), *Bacteroides fragilis* (n = 1), *Salmonella* spp. (n = 1), *Haemophilus influenzae* (n = 1), *Candida parapsilosis* (n = 1).

d 1 positive blood culture set out of 2 sets with Gram-positive microorganisms indicated by microbiology lab.

Median time to BCID2 review was significantly reduced in the post-cohort compared to the pre-cohort (3.6 hours vs 9.3 hours, *P* < .01). A higher proportion of patients had documented interventions in the post-cohort versus the pre-cohort (51.3% vs 39.1%, *P* = .011). Acceptance rates of antimicrobial interventions were not significantly different between the groups: 78.1% in the post-cohort compared to 87.5% in the pre-cohort (*P* = .218).

Among accepted antimicrobial interventions, time to antimicrobial order changes was also reduced in the post-cohort (0.1 hours vs 1.2 hours, *P* = .05). Table 2 summarizes types of accepted antimicrobial order changes and additional secondary outcomes. No statistical differences were observed in empiric antimicrobial concordance with final pathogen susceptibilities, 30-day mortality, or 30-day readmission.

**Table 2.** Primary and secondary outcomes

Outcomes	Pre-cohort (n = 192)	Post-cohort (n = 230)	*P*-Value
Time to BCID2 review (hours), median (IQR)	9.3 (6.6, 15)	3.6 (1.5, 9)	**< .01**
Interventions documented, n (%)	75 (39.1)	118 (51.3)	**.011**
Types of interventions, n (%)			
* Staphylococcus aureus* bacteremia consult^a^, n (%)	29 (38.7)	32 (27.1)	.093
Recommended ID consult, n (%)	6 (8.0)	13 (11.0)	.493
Antimicrobial interventions, n (%)	40 (53.3)	73 (61.9)	.241
Accepted, n (%)	35 (87.5)	57 (78.1)	.218
Time to accepted antimicrobial order change (hours), median (IQR)	1.2 (0.1, 7.9)	0.1 (0, 2.2)	.05
Types of accepted antimicrobial order change^b^, n			.346
Initiation, n (%)	3/35 (8.6)	6/57 (10.5)	
De-escalation/Discontinuation, n (%)	29/35 (82.8)	41/57 (72.0)	
Escalation, n (%)	3/35 (8.6)	10/57 (17.5)	
Final susceptibilities of pathogenic organisms concordant with empiric antimicrobial therapy^c^, n (%)	123 (96.9)	162 (96.4)	.164
30-day all-cause mortality, n (%)	39 (20.3)	59 (25.7)	.196
30-day all-cause readmission, n (%)	28 (14.6)	41 (17.8)	.370

Abbreviations: BCID2, Blood Culture Identification 2; ID, infectious diseases; IQR, interquartile range.

a *Staphylococcus aureus* bacteremia consults include *Staphylococcus aureus* detected in polymicrobial BCID2 results (Pre-cohort, n = 5; Post-cohort, n = 6) and *Staphylococcus aureus* alone detected by BCID2 (Pre-cohort, n = 24; Post-cohort, n = 26).

b Initiation = starting an antimicrobial with activity against the pathogen detected by BCID2; De-escalation/Discontinuation = narrowing spectrum of activity or stopping of therapy; Escalation = broadening spectrum of activity of therapy with patients already initiated on antimicrobials.

c Pre-cohort = 127 pathogenic organisms; Post-cohort = 168 pathogenic organisms; Susceptibilities were not performed on contaminants.

## Discussion

We evaluated the impact of adding overnight pharmacist review of BCID2 results to a typical ASP workflow demonstrating an increase in interventions as well as reduction in time to review and intervention. While current literature focuses primarily on ID and ASP review of rapid diagnostics, our study highlights a novel expansion of these activities utilizing ICU pharmacists to act as ASP extenders by implementing prospective audit and feedback in concordance with ASP guidance. The decreased time to antimicrobial order changes was likely due to earlier review of BCID2 results and communication of recommendations with ICU providers via telephone or secure private messaging.

Furthermore, the significantly higher number of documented interventions post-implementation demonstrated the impact of more rapid reviews, which allowed for faster changes in antimicrobials. There were numerically lower acceptance rates of overnight ICU pharmacist recommendations, which may be the result of intervention timing with cross-covering overnight care teams being less willing to make changes without primary team consideration. However, the ASP team created and implemented clear BCID2 guidance for ICU pharmacists to ensure appropriate institutional-based recommendations.

In patients with accepted antimicrobial interventions, the overnight ICU pharmacist workflow (Supplemental Figure 2) was associated with a reduced median subsequent time to antimicrobial change. These results further support that earlier review of rapid diagnostics by non-ASP clinicians have the ability to decrease time to initiation of effective therapy.^7,8^ Among patients who warranted initiation or escalation of antibiotics, this is a significant finding, as delaying effective antimicrobial therapy for bloodstream infections has been associated with increased mortality.^9^ Although there was no significant difference in the types of accepted antimicrobial order changes, there was a higher percentage of initiations and escalations of therapy within the post-cohort.

Additional strengths include our definitions of antimicrobial order changes being mostly aligned with previous studies.^7,10^ However, the lack of concordance in definitions and differentiation between spectrum of activity and exposure to more than one antimicrobial complicates these antimicrobial therapy definitions. Furthermore, a high percentage of concordance of final susceptibilities with empiric therapy was maintained in the post-cohort demonstrating our ICU pharmacists’ recommendations were appropriate based on our institutional ASP BCID2 guidance and local antibiogram patterns.

Limitations include its single-center, retrospective, pre-post study design. Only overnight BCID2 results for ICU patients were reviewed, so future studies are warranted to explore the impact of a similar intervention in non-ICU settings. An additional limitation was the potential for missing intervention documentation. As a result of this project, we completed additional education on the overnight ICU pharmacist workflow (Supplemental Figure 2) with emphasis on review/intervention documentation. Finally, implementation of overnight ICU BCID2 review included the first month of our post-group cohort which may have introduced bias.

Our study demonstrates the benefit of rapid diagnostics in combination with overnight ICU pharmacy services to improve time to stewardship intervention. Our findings emphasize the importance of integrating rapid diagnostics and continuous ASP review, specifically in the ICU, to optimize the management of bloodstream infections.

## Supplementary material

For supplementary material accompanying this paper visit http://doi.org/10.1017/ash.2025.28. click here to view supplementary material

## Supporting information

Arduser Sindelar et al. supplementary material Arduser Sindelar et al. supplementary material
