# Supplementary material for: Stewarding beyond the 9–5: Implementation of overnight review of rapid blood culture identification panel results by intensive care unit pharmacists
Source: Antimicrob Steward Healthc Epidemiol. 2025 Feb 17;5(1):e52. doi: 10.1017/ash.2025.28 (PMC11869055; doi:10.1017/ash.2025.28)
Supplement: Supplementary file 1 [file S2732494X25000282sup001.docx]

**Stewarding Beyond the 9 to 5: Implementation of Overnight Review of Rapid Blood Culture Identification Panel Results by Intensive Care Unit Pharmacists**

**Supplemental Material**

**Supplemental Figure 1: Patient Cohorts**

**
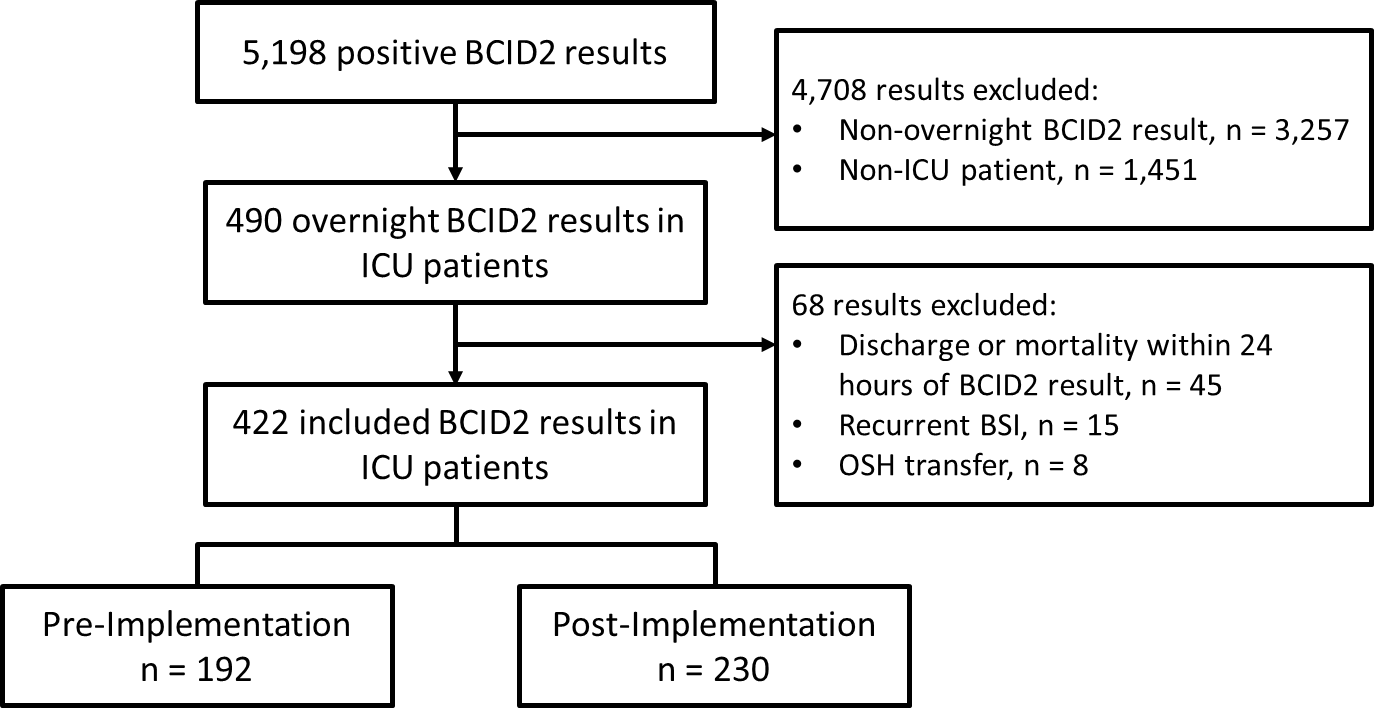
**

**Abbreviations: BCID, blood culture identification; ICU, intensive care unit; BSI = bloodstream infection; OSH, outside hospital**

**Supplemental Figure 2: Overnight ICU Pharmacist Review Workflow**

Pharmacist notifies team with results and recommendations for adjusting therapy

Pharmacist documents intervention in ASP monitoring review and i-Vent

Pharmacist documents review in the ASP monitoring review and i-Vent

No

Yes

Is there an opportunity for an antimicrobial change?

Positive BCID2 results in chart

ICU pharmacist reviews BCID2 results and evaluates current antimicrobial therapy using Nebraska Medicine’s BCID interpretation and treatment guidance

**Abbreviations: BCID, blood culture identification; ICU, intensive care unit; ASP, antimicrobial stewardship program**

**Supplemental Figure 3: Documented Interventions**

**
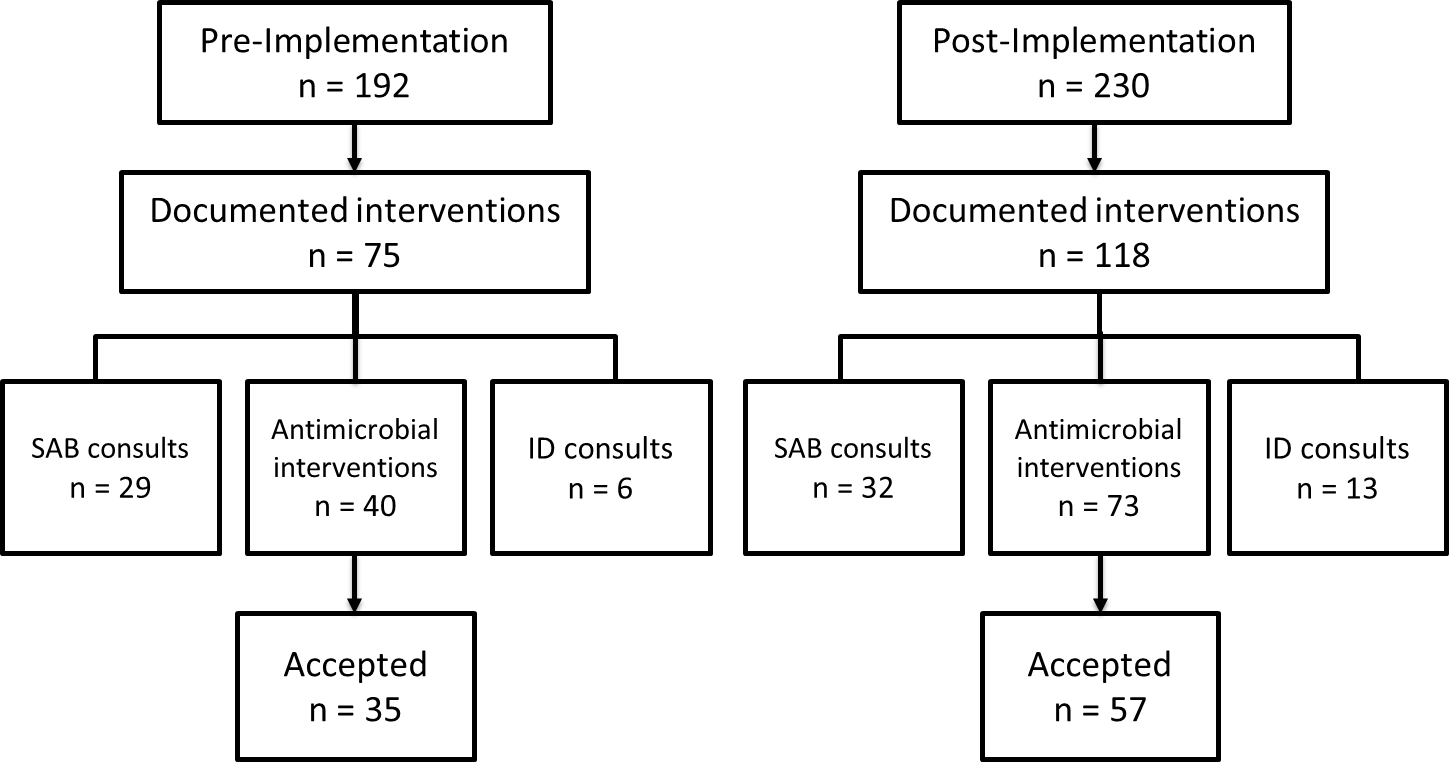
**

**Abbreviations: SAB, *Staphylococcus aureus* bacteremia; ID, infectious diseases**

**Nebraska Medical Center Recommendations for Use of BCID2**

[**https://www.unmc.edu/intmed/_documents/id/asp/news/bcid2-final-8-11-21.pdf**](https://www.unmc.edu/intmed/_documents/id/asp/news/bcid2-final-8-11-21.pdf)

**Nebraska Medicine Overnight ICU Pharmacist Review Standard Operating Procedure**

[**https://www.unmc.edu/intmed/_documents/id/asp/clinicmicroovernighticu_bcidreview_sop_v3.pdf**](https://www.unmc.edu/intmed/_documents/id/asp/clinicmicroovernighticu_bcidreview_sop_v3.pdf)

**Supplemental Figure 4: Antimicrobial Stewardship Program BCID2 Review Workflow**

**Pre-cohort Antimicrobial Stewardship Program (ASP) review consisted of daily review of Blood Culture Identification 2 (BCID2) results between the hours of 8:00 am to 5:00 pm by ASP pharmacists and physicians.**

ASP pharmacist or physician reviews BCID2 results and evaluates current antimicrobial therapy using Nebraska Medicine’s BCID interpretation and treatment guidance

Positive BCID2 results in chart

Does the patient need an intervention?

ASP pharmacist or physician notifies team with recommendations for adjusting therapy and documents recommendations in ASP antimicrobial monitoring review

ASP pharmacist or physician documents review in ASP antimicrobial monitoring review

No

Yes
